# Direct electrophoretic microRNA preparation from clinical samples using nanofilter membrane

**DOI:** 10.1186/s40580-019-0212-3

**Published:** 2020-01-13

**Authors:** Kidan Lee, Jae-Hyun Kang, Hyun-Mi Kim, Junhyoung Ahn, Hyungjun Lim, JaeJong Lee, Wan-Jin Jeon, Jae-Hoon Lee, Ki-Bum Kim

**Affiliations:** 1 Department of Materials Science and Engineering, Seoul National University, Seoul, 08826 Republic of Korea; 2 Research Institute of Advanced Materials, Seoul National University, Seoul, 08826 Republic of Korea; 3 Department of Nano Manufacturing Technology, Nano Convergence Mechanical Systems Research Division, Korea Institute of Machinery & Materials (KIMM), Daejeon, 34103 Republic of Korea; 4 Department of Nanomechatronics, University of Science & Technology, Daejeon, 34113 Republic of Korea; 5 Heimbiotek Inc., Seongnam, Gyeonggi-do 13486 Republic of Korea

**Keywords:** Nucleic acid extraction, Nucleic acid preparation, miRNA extraction, Nanofiltration, Nanoporous membrane, Liquid biopsy

## Abstract

A method to directly collect negatively charged nucleic acids, such as DNA and RNA, in the biosamples simply by applying an electric field in between the sample and collection buffer separated by the nanofilter membrane is proposed. The nanofilter membrane was made of low-stress silicon nitride with a thickness of 100 nm, and multiple pores were perforated in a highly arranged pattern using nanoimprint technology with a pore size of 200 nm and a pore density of 7.22 × 10^8^/cm^2^. The electrophoretic transport of hsa-mir-93-5p across the membrane was confirmed in pure microRNA (miRNA) mimic solution using quantitative reverse transcription-polymerase chain reactions (qRT-PCR). Consistency of the collected miRNA quantity, stability of the system during the experiment, and yield and purity of the prepared sample were discussed in detail to validate the effectiveness of the electrical protocol. Finally, in order to check the applicability of this method to clinical samples, liquid biopsy process was demonstrated by evaluating the miRNA levels in sera of hepatocellular carcinoma patients and healthy controls. This efficient system proposed a simple, physical idea in preparation of nucleic acid from biosamples, and demonstrated its compatibility to biological downstream applications such as qRT-PCR as the conventional nucleic acid extraction protocols.

## Introduction

In genetic analysis of clinical samples, nucleic acid extraction from raw biosamples is the initial pre-treatment step to purify and concentrate the nucleic acid from other constituents of the samples including proteins, lipids, and other organic molecules. One of the mainstream solid-phase extraction techniques is based on the charge interaction principle, using a surface modified binding media such as silica bead (or membrane) or magnetic bead to selectively collect the nucleic acid from the sample and ethanol-based buffers to wash out impurities [1–5]. While this method and related products are well established [6–9], it normally constitutes more than 10 steps, thus requires substantial time and skilled personnel to produce stable extraction results. In addition, apparatus such as centrifuge and heating blocks should be used in the chemical processes to lyse the cells, separate the nucleic acids, and clear away the unnecessary organic residues [3, 6]. Consequently, the nucleic acid extraction protocols spatially limit the genetic analysis to a laboratory procedure, making it difficult to expand to quick and handy on-site detection of the target gene in biological or clinical samples [3].

The complexity of the conventional nucleic acid extraction method led to the introduction of alternative techniques, which have focused on simplifying the process and reducing the extraction time [4, 6]. Among the reported strategies, lab-on-a-chip approaches targeted systemization of the procedure by integrating the extraction column, binding media, and buffers in a single device [5, 6, 10–12]. In typical lab-on-a-chip experiments, a single microfluidic chip per extraction and small amounts of buffers were used to isolate DNA or RNA in a relatively short time (~ 30 min in general) [13–19]. These easy processes provided comparable quality of purified nucleic acids to those of the conventional protocols in terms of polymerase chain reaction (PCR) amplification or gene sequencing results [15, 16, 18].

Alternatively, a lab-on-a-chip protocol developed by Heller group employed the physical principle of dielectrophoresis to isolate cell-free DNA in a ~ 10 min of time from blood samples [19–21]. In this physical method, an alternative current (AC) electric field was delivered to the chip via embedded platinum electrodes, creating local electric field divergence [22]. The spatially non-uniform electric field and the polarizability differences between the biomolecules in blood and the medium generated a dielectrophoretic force. Under the AC field with ~ 10 V of voltage and kHz-order frequency, the highly charged DNA and other less or non-charged particles in blood were dielectrophoretically dragged in the opposite direction to each other [21]. The consequent PCR band intensities of the isolated DNA were at a comparable level to those from the conventional columnar kits [22]. In summary, the lab-on-a-chip methods improved the conventional nucleic acid extraction protocol in terms of process simplicity and time, still demonstrating competitive extraction performances. Nevertheless, the methods still consisted of multiple steps of mainly sequential injection of buffers and required heavy apparatus such as a liquid injector [15], a centrifuge [14, 18], or a high AC field generator [21, 22] for the dielectrophoretic DNA separation chip.

In this study, we developed a simpler nucleic acid preparation protocol in which direct current (DC) electric field drew the highly charged DNA or RNA from positively charged and weakly or non-charged species in biological and clinical samples. Additionally, for separation, a ~ 100-nm thick fabricated nanoporous membrane was inserted to prevent mixing of the sample and collection buffers and to block larger debris in the sample from transporting to the collection buffer. By its simple electrophoresis principle, this method only required a membrane chip, chambers, a pair of electrodes, and a DC power supply, where the last can ultimately be replaced by batteries used in households to create a compact nucleic acid preparation system even suitable for on-site operation.

We demonstrated the proof-of-concept experiment of the direct electrophoretic nucleic acid preparation and that this physical principle and method were compatible with popular downstream applications such as PCR [23]. After optimizing the experimental parameters including operation time and voltage, we performed the nucleic acid preparation from the human blood sera. Cell-free nucleic acid isolation from blood serum and its analysis are a part of liquid biopsy: a new, non-invasive, and cost-effective cancer prognosis and diagnosis technique in which cancer type and stage are identified using tumor-related genes existing in blood [24–27]. In particular, the target nucleic acid in this work was microRNA (miRNA), a non-coding gene [28] that regulates the gene expression [23] and the tumor growth [29, 30], which is relatively complicated to isolate from the blood due to its small molecular size [31]. The goal of this work was to introduce an easy nucleic acid preparation principle and to show that the electrical process worked effectively in a challenging application, collecting small miRNA from clinical samples. A commercial columnar extraction kit was selected as a reference to evaluate the miRNA preparation performances of the electrical system, where the extracted gene levels were compared using quantitative reserve transcription PCR (qRT-PCR).

## Experimental

### Chemicals and reagents

All chemicals and reagents were used as received without further purification. Mature hsa-miR93-5p mimic was purchased from Genolution (Seoul, Korea), and tris–EDTA (TE) buffer (1×, pH 8.0, RNase and DNase free) and chloroform were purchased from Sigma Aldrich (St. Louis, USA). qRT-PCR was performed using miScript II RT Kit, miScript SYBR Green PCR Kit, and miScript Primer Assay (hsa-miR93-5p primers) from Qiagen (Hilden, Germany). miRNeasy Serum/Plasma Kit from Qiagen was used for the chemical extraction of miRNA from the sera. Agarose gel, tris acetate-EDTA (TAE) buffer, 25/100 bp mixed DNA ladder (all from Bioneer Inc., Daejeon, Korea), and gel loading buffer (Bionics, Seoul, Korea) were purchased to carry out gel electrophoresis. All reagents other than TE and TAE buffer, chloroform, agarose gel, gel loading buffer (all at room temperature), miRNeasy kit (2–4 °C), and the clinical serum samples (− 80 °C) were stored at − 20 °C upon receipt. Polydimethysiloxane (PDMS) monomer and curing agent were purchased from The Dow Chemical Company (Michigan, USA), and stored at room temperature.

### Nanofilter membrane fabrication

The fabrication procedure started from low-pressure chemical vapor deposition (LPCVD) of 500-nm low-stress silicon nitride (SiN_x_) onto a double-side polished 4-in. silicon (Si) wafer. Nanoimprint resist (poly(urethane acrylate)) was coated on one side of the wafer, where the nanopore pattern of 200-nm-large nanopores with 400-nm center-to-center distances was imprinted using pre-made nanoimprint mold [32]. The nanopores were pre-defined by partial etch of SiN_x_ in ~ 100 nm depth using inductively coupled plasma (ICP) etcher (Oxford Instruments PlasmaPro System100 Cobra), followed by the creation of the freestanding membrane according to the conventional solid-state nanopore fabrication protocol [32]. The imprinted pattern should be etched before the membrane fabrication step, and at the same time, the SiN_x_ film should be free of penetrating pores to effectively serve as the Si wet etch mask. As the last step, the nanopores were fully perforated by partial dry etch of SiN_x_ from the backside of the predefined area. The membrane thickness was determined by measuring the SiN_x_ thickness of the backside of the supporting chip using Nanospec® (Nanometrics Inc.).

### Electrical preparation system setup

Two compartments of custom-made Teflon® flow cells, two 1 cm × 1 cm × 2 mm PDMS panels with a 3-mm large hole punched at the center, and the nanofilter membrane device were assembled using a pair of screws. To construct the PDMS blocks, the elastomer base and the curing agent were mixed in a 10:1 weight ratio and the mixture was cured at room temperature overnight. The volumes of the solutions loaded in the reservoir and the collection chambers were 150 µl and 75 µl respectively in all nanofilter experiments. Pt electrodes connected to a DC power supply (Keithley® 237 instrument) were immersed in each chamber. The chambers were reused after cleaning with fresh piranha solution for 10 min and rinsing with deionized water for 10–15 min. Pt electrodes were cleaned after each run with 70% ethanol and deionized water and dried before use.

### miRNA preparation experiments

Before all experiments, clean TE buffer was loaded in both chambers and 10 V was applied to either chamber to check if the ionic path had been effectively created through the nanopores. Then, 150 µl of 100 pg/µl miR93-5p mimic in TE buffer (reservoir chamber, input) and 75 µl of bare TE buffer (collection chamber, output) were injected to verify the miRNA transport. The applied voltage to the collection chamber was 0, 1, 2, 5, and 10 V, and each run lasted for 30 min. As above, miRNA was driven from the clinical sera of 150 µl volume to 75 µl TE buffer. For all serum miRNA collection experiments, 2 V was applied for 30 min. The same 150 µl sera were used for the chemical extraction using the purchased kit, basically following the manufacturer’s protocol except for the last elution step: RNase-free water of 75 µl was used instead of the suggested volume of 14 µl. All miRNA preparations were conducted for no less than 3 times for the same sample, but the kit extractions from the clinical sera were only performed once for each sample due to the limited supply of the sera (~ 700 µl for each donor). The clinical serum samples used in this work were provided by the National Biobank of Korea. The experiments using the human blood serum of hepatocellular carcinoma patients and healthy individuals were approved by the institutional review board of Seoul National University (IRB No. E1804/003-004, 2018-04-16).

### qRT-PCR, gel electrophoresis, and spectrophotometry analysis

The reverse transcription (RT) reaction was performed no later than a day after the miRNA was collected electrically or chemically. The synthesized complementary DNA (cDNA) was stored at − 20 °C before qPCR experiments. RT and qPCR processes all followed the protocol provided by the kit manufacturer and were performed using Applied Biosystems® 2720 Thermal Cycler (RT) and Bio-Rad® CFX384 Touch™ Real-Time PCR Detection System (qPCR). RT reaction was completed after two-step incubations under 37 °C for 60 min and 95 °C for 5 min. For the cDNA, 45 qPCR cycles (94 °C, 15 s—55 °C, 30 s—70 °C, 30 s) were performed after the initial activation step of 95 °C incubation for 15 min. Gel electrophoresis and imaging were carried out using Mupid® 2-Plus electrophoresis system (Advance, Japan) and Gel Doc™ XR + system (Bio-rad, USA). The spectrophotometry measurements were conducted using Nanodrop® 2000 from Thermo Fisher Scientific®.

## Result and discussion

### System setup and fabrication of nanofilter membrane device

**Fig. 1:**
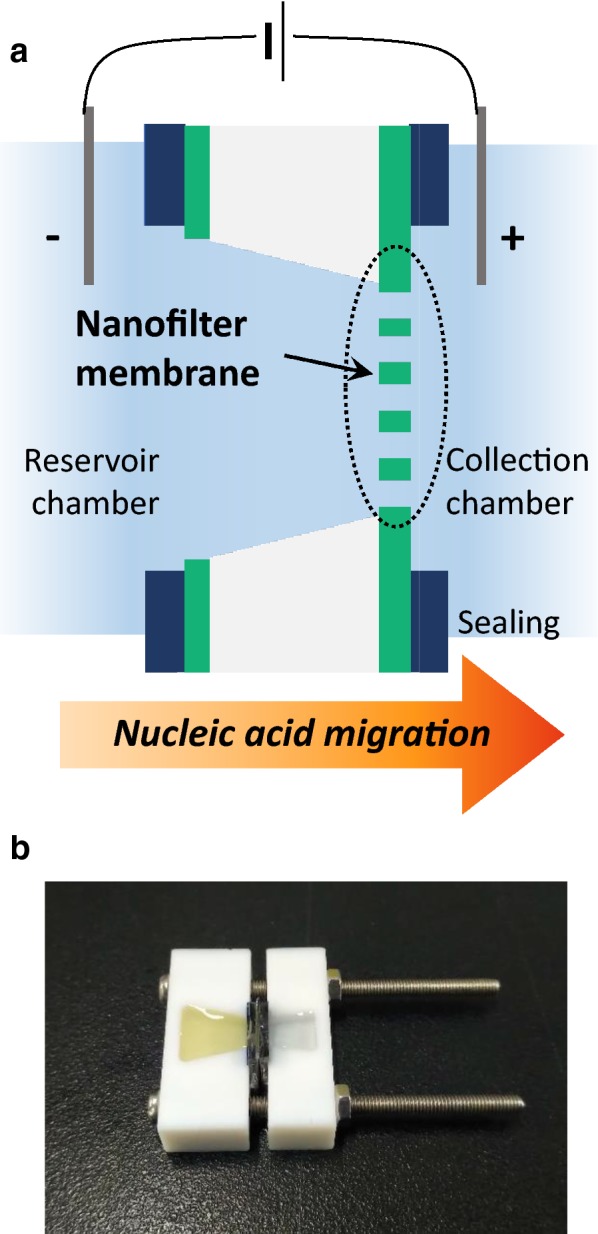
Direct electrophoretic nucleic acid preparation schematics. **a** A model image of the electrophoretic nucleic acid preparation system. This image is not in scale. **b** A photograph of the assembled direct preparation cell, with serum and TE buffer inserted in the reservoir chamber (left chamber) and the collection chamber (right chamber) each

The schematics and the actual setup of the direct electrophoretic nucleic acid preparation using the nanofilter membrane device were displayed in Fig. 1. The principle of the electrophoretic preparation was to simply drive the negatively charged nucleic acid in the reservoir chamber (sample) to the collection chamber (buffer), where the positive voltage was applied via external DC power source (Fig. 1a). To construct the electrical preparation system, the membrane and the chambers were assembled with elastomer gaskets on each side of the chambers to prevent leakage of the liquids (Fig. 1b). The sample and the collection buffer (1× TE buffer in this work) were injected into each chamber, and a pair of electrodes was loaded. After setup, the nanopores acted as the separation layer between the chambers and the only transport path for the ions, the fluid, and the biomolecules.

The nanofilter membrane should prevent the mixing of the buffers in the two chambers while facilitating the transport of the nucleic acid molecules. In the molecular transport perspective, conventional molecular filter membranes such as track-etched polycarbonate membrane were unfavorable to use in this work because their 10 µm-order thicknesses resulted in low molecular fluxes [33]. In contrast, the membranes of sub-micron thicknesses were advantageous in promoting the molecular transport to the opposite chamber, while the thinness may sacrifice the mechanical vulnerability of the membranes.

Consequently, to create a thin and robust nanoporous membrane, silicon nitride was selected as the membrane material and thus semiconductor fabrication technique as the device fabrication method. From its excellent mechanical robustness and chemical stability, SiN_x_ thin film has been widely used in solid-state nanopore fabrication [34, 35]. The semiconductor fabrication technology, lithography in particular, has a strong advantage in forming a highly aligned structure with designed feature size [36, 37]. This allowed the fabrication of a packed, orderly network of identical pores.

**Fig. 2:**
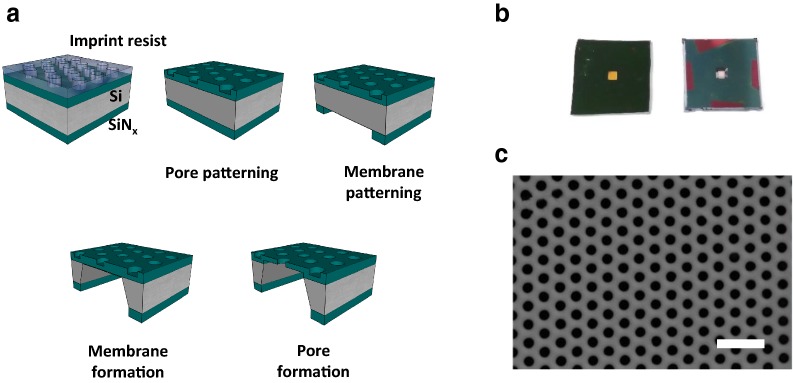
Nanofilter membrane fabrication. **a** Fabrication steps of the nanofilter membrane device. The image is not in scale. **b** A photograph of the nanofilter membrane device, the front side (left) and the backside (right). The square membrane is located at the center, having 700 μm width. The size of the chips is 1 cm × 1 cm. **c** A scanning electron microscope (SEM) image of the uniform arrangement of the nanopores in the nanofilter membrane. The scale bar indicates 1 μm

Figure 2 illustrated the fabrication procedure of the nanofilter membrane device with the images of the completed devices and the porous membrane [32, 34, 35, 38, 39]. Figure 2a presented the nanofilter membrane fabrication sequence, explained in detail in the experimental section. Nanoimprint technique is an efficient lithographic method that features a stamping process of a reusable polymeric mold onto nanoimprint resist, reducing time and cost for the fabrication of nanostructures [32]. As the result, Fig. 2b, c clearly displayed that the nanofilter membrane consisted of well-aligned and uniform nanopores of 200-nm diameter was formed at the center of a 1 cm × 1 cm chip. The nanopore density calculated from Fig. 2c was 7.22 × 10^8^/cm^2^.

In determining the dimensions of the pore, the membrane, and the device, the reproducibility in fabrication, as well as the separation principle, were considered. Since the operation principle was mainly based on the electrical charge and the size of the particles, nanopores should be small enough to sieve relatively large-sized impurities and large enough to allow the flux of small particles including nucleic acids [33]. To stably produce a well-arranged array of small nanopores of nanometer-level size, 200 nm was selected as the optimum pore diameter. Nanoporous membranes with smaller pore sizes have been reported in a number of articles [40–42]; however, the distinct feature of the nanofilter membrane in this work was the ordered network of uniform pores spanning in a whole membrane to increase the channel area for the biomolecular transport. The area (700 μm to 1 mm in width) and the thickness (~ 100 nm) of the membrane were determined upon its mechanical stability during the electrical preparation experiments. During the device fabrication, the thickness of the membrane was indirectly monitored by measuring the SiN_x_ thickness of the partially etched backside of the device using ellipsometry. Finally, the chip size was designed for the convenient handling of the device.

### Direct and electrophoretic miRNA preparation: a proof-of-concept

First, to validate that the nucleic acid can electrically transport and be collected across the nanofilter membrane, hsa-mir-93-5p (miR93-5p) mimic was electrophoretically drawn from the pure miRNA mimic solution with a known concentration of 100 pg/µl. In this experiment, 1–10 V was applied to the collection chamber for 30 min to drag the negatively charged miRNA in the reservoir chamber across the membrane. Voltage application time was first set as above referring to the previous reports on lab-on-a-chip nucleic acid extraction protocols [13–18]. Longer operation times would allow more miRNA to transport to the collection chamber, but the buffers started to dry significantly after 30 min, limiting the processing time up to half an hour. Closed packaging would enable long-term and stable runs, yet still shorter times would be preferable in terms of efficiency as long as the collected miRNA is analyzable in downstream applications. The input sample volume (150 µl in the reservoir chamber) was attributed to the commercial columnar-based methods, a reference to assess the performance and efficiency of the direct electrophoretic preparation system. Following the input volume, the output volume was set to 75 μl for stability that the collection buffer stayed intact for 30 min.

The collected miRNA was amplified using qRT-PCR to compare the miRNA collection efficiency under each applied voltage condition. Threshold cycle (C_t_) values were obtained in all voltages, indicating that the miRNA has transported across the nanofilter membrane under the bias voltage. When the same setup was left for 30 min without bias voltage to assess the diffusive contribution to the transport, the miRNA concentration in the collection chamber was less than 5% of those collected under the electrically biased conditions. This result suggested that the electric field generated by the applied voltage was indeed the major driving force for the miRNA movement.

**Fig. 3:**
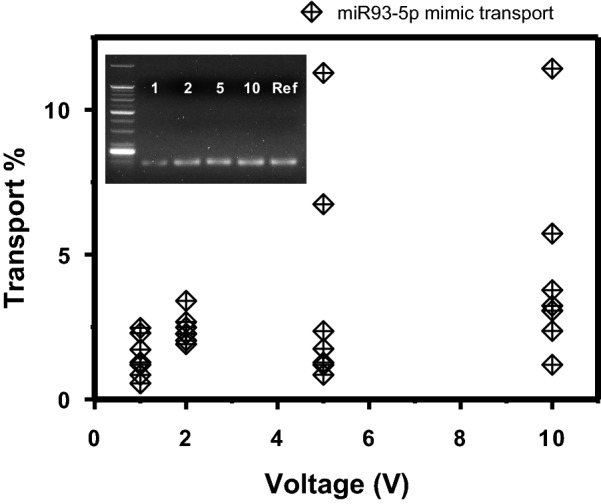
Electrophoretic transport of miRNA across the nanofilter membrane. Each diamond point indicates the electrophoretically transported quantity of miRNA for 30 min relative to the input in each run (*n* = 7 for each voltage). Inset is a gel image resulting from gel electrophoresis of the amplified genes. The numbers labeled in the inset indicate the applied voltages in V. The positive control band was labeled as ‘Ref’

To compare the molecular transport by the applied voltage and optimize the voltage condition, the collected quantities of miRNA relative to the input were calculated from the C_t_ values and the buffer volumes. Figure 3 presented the transported % of miRNA under 1, 2, 5, 10 V. Two regions are identifiable in the graph; 1 V and 2 V with lower percentages but clustered data points, and 5 V and 10 V with a couple of points above 5% but scattered distributions. The data fluctuation at 5 V and 10 V may be attributed to uneven evaporation and migration of the fluid under these conditions, which will be discussed below with graphics. Considering performance reproducibility, the lower voltages, especially 2 V, were the favorable collection voltage in this work. Additionally, the molecular stability of the collected miRNA was confirmed in gel electrophoresis, where the band positions obtained from the collected miRNAs corresponded to that from the reference miRNA mimic. In conclusion, from the preliminary experiments using a known miRNA mimic solution, the idea of direct electrophoretic nucleic acid preparation was practically validated in consecutive qRT-PCR. Applying 2 V for 30 min was suitable in terms of quantitative consistency over multiple experiments. Under this condition, the average collection rate of miRNA from the pure miRNA solution was 12.2 pg/min.

### System stability in the electrophoretic preparation setup

**Fig. 4:**
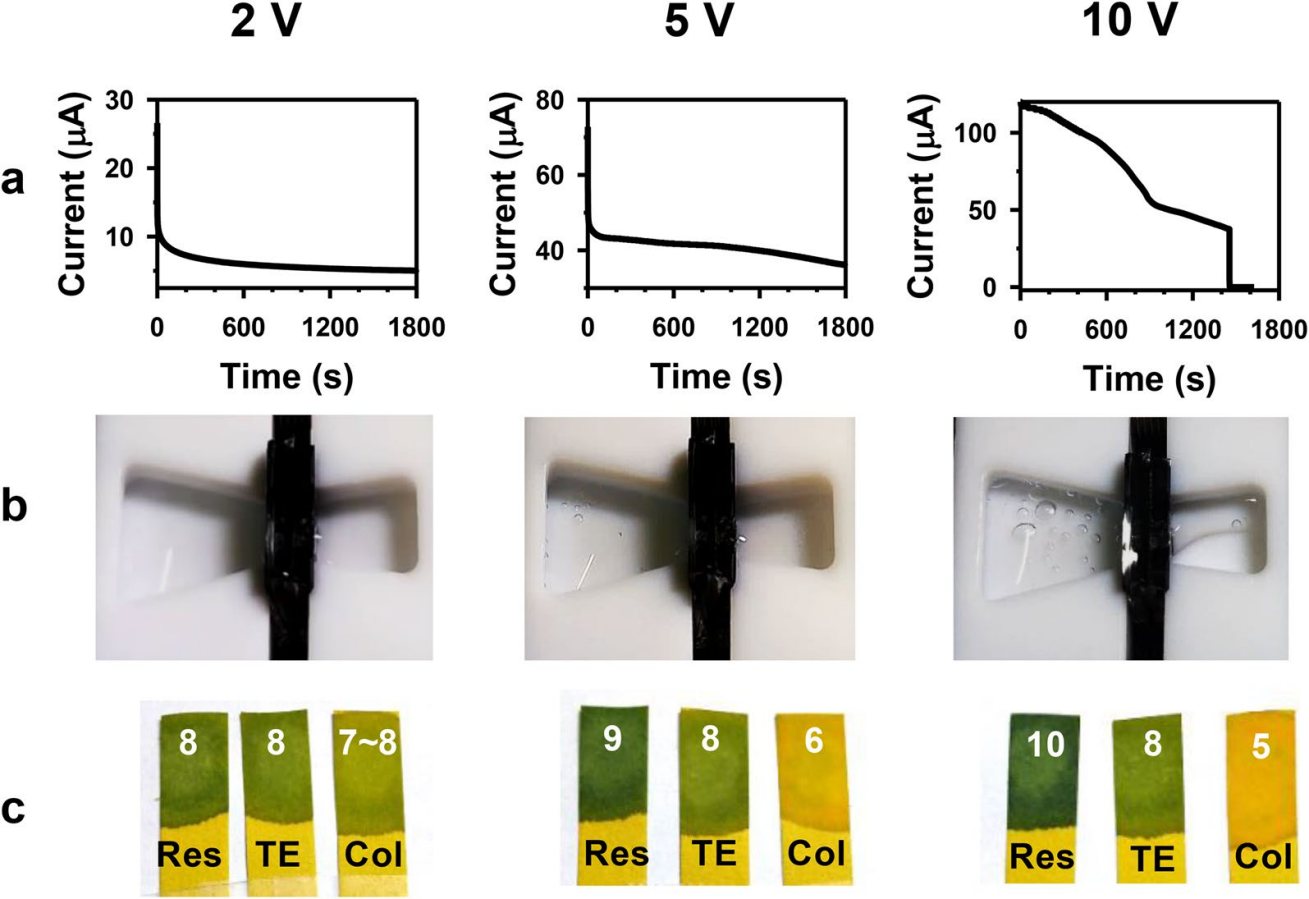
Stability during and after the electrophoretic preparation experiments. **a** Ionic currents during 30 min, **b** the chambers after 30 min, and **c** pH changes of solutions in the reservoir chamber (Res) and the collection chamber (Col) after 30-min experiments with the initial buffer pH (TE) under 2, 5, 10 V conditions. The measured pH values were labeled on each pH meter paper strip

As well as the consistency in data, electrical and electrochemical stability of the system during the experiment should be considered for optimizing the collection voltage. Ionic current during 30 min and change in the chamber and buffer pH after 30-min experiments were presented by the applied voltage (Fig. 4). In Fig. 4a, the ionic current stayed stable after a capacitive delay at 2 V, but the current curve started to decrease after 1200 s (5 V) and ~ 200 s (10 V) at the higher voltages.

The reducing ionic current would have resulted from the decrease in the collection buffer volumes under higher voltages; the buffer partly evaporated and migrated to the reservoir chamber. Figure 4b clearly showed that the collection buffer was significantly depleted after 30-min application of 10 V. Under 5 V, though hardly noticeable in the figure, about 20–30% of the collection buffer in average moved to the opposite chamber during 30 min while it was steady under 1–2 V. The direction of the fluid movement was the same as the electroosmotic flow direction expected from the movement of the positive counterion gathered near the negatively charged SiN_x_ surface at pH 8.0 [43]. By principle, electroosmotic flow inside the nanopores existed also under lower voltages, but the changes in the buffer volumes were minimal in these conditions presumably due to the lower electric field.

In addition to the change in the collection buffer quantity, air bubbles could be found in the chambers at 5 V and 10 V. As Pt electrodes were used, the evolved bubbles would be hydrogen and oxygen generated from water electrolysis reaction. The gas evolutions were indirectly identifiable through pH alterations of the buffers after 30 min (Fig. 4c). The direction of pH change corresponded to the water electrolysis reactions in a basic solution (pH of TE buffer: 8.0) shown below:

$$\left( {{\text{cathode}}} \right){\text{ 4H}}_{{2}} {\text{O }} + {\text{ 4e}}^{ - } \to {\text{2H}}_{{2}} + {\text{ 4OH}}^{ - } \left( {\text{pH increase}} \right),$$

$$\left( {{\text{anode}}} \right){\text{ 4OH}}^{ - } \to {\text{O}}_{{2}} + {\text{ H}}_{{2}} {\text{O }} + {\text{ 4e}}^{ - } \left( {\text{pH decrease}} \right).$$

The standard potential (1 M, 25 °C, 1 atm) of this reaction is 1.23 V. Under 2 V, although the reaction would also have been present, there was a minimal effect in disrupting the buffering capability according to Fig. 4c. The migration of fluid, the air bubble generations, and the pH change in solutions after 30-min runs were observed in all 5 and 10-V runs. Therefore, the electrically induced fluid migration and water electrolysis reaction were active under the high voltages, and the instability of the system would have adversely affected the reproducibility of the transport yield. To summarize, considering the quantitative and qualitative discussions above—though miRNA was reported to be stable under a wide range of pH [44]—2 V was set as the optimum voltage condition for the electrophoretic miRNA collection. Henceforth, all experiments were conducted under 2 V for 30 min in the sections below.

### Yield and quality of the electrically collected miRNA

**Fig. 5:**
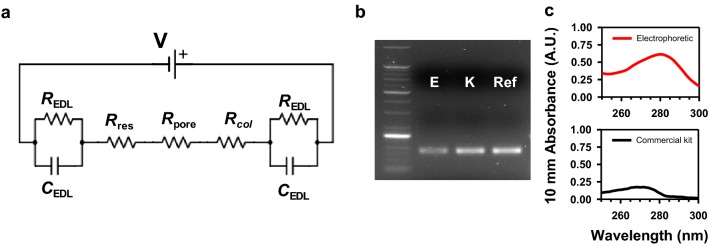
Working principles of the electrophoretic miRNA preparation method. **a** A simple circuit model of the electrophoretic system. Each electrical component is explained in the text. **b** Gel image of the miRNA from human blood serum separated using the electrophoretic protocol (E) and the commercial kit (K), and the positive control (Ref). **c** UV–Vis spectrophotometry result of the electrophoretically collected (upper, red line) and the chemically extracted (lower, black line) miRNA. The y-axis represents the absorbance at 10 mm pathlength

Even though miRNA successfully and stably transported across the nanofilter membrane as above, the electrically collected amount of miRNA was as small as a few percent of the input quantity (Fig. 3). To analyze the origin of the migration yield or the electrophoretic driving force, the chamber system was constructed as a simple circuit model in Fig. 5a. The circuit consisted of a power source (V), resistances of the fluidic chambers (*R*_res_, *R*_col_) and the nanopores (*R*_pore_), and the resistive and capacitive components in the electric double layer (EDL) at the electrode-solution interface (*R*_EDL_, *C*_EDL_) [45]. Here, the capacitance of the silicon chip was neglected from its small value (in pF order) [46, 47] and for simplicity. From the nonzero saturation current at 2 V in Fig. 4a, the platinum electrodes were assumed a realistic electrode, having the resistive term *R*_EDL_ [48]. In principle, the electrical driving force was proportional to the electric field thus the voltage drops in each part of the system: more fundamentally, the relative magnitude of each resistance.

To quantitatively identify each component in the circuit, the ionic current curve was recalled from Fig. 4a. At 2 V, the time-dependent ionic current can be mathematically interpreted as in the equation below:

$$I\left(t\right)=\left\{I\left(0\right)-I\left(\infty \right)\right\}\cdot {\mathrm{e}}^{-\left(\frac{1}{{R}_{EDL}{C}_{EDL}}+\frac{1}{\left({R}_{res}+{R}_{col}+{R}_{pore}\right){C}_{EDL}}\right)\cdot t}+I\left(\infty \right),$$

where *I*(*t*) is the ionic current at 2 V as a function of time *t*, *I*(0) is the initial ionic current at *t* = 0, and *I*(∞) is the saturated ionic current [46]. By curve fitting and calculating the chamber and the nanopore resistance from the known dimensions, total EDL resistance was 1.13 × 10^5^ Ω, *R*_res_ + *R*_col_ was 1.23 × 10^4^ Ω, and *R*_pore_ was 39.8 Ω.

Calculating from the resistances, nearly 90% of the total voltage drop occurred at the electrode-buffer interfaces. Pt electrodes were ideal in the electrochemical aspect—the byproducts of the electrochemical reaction were only hydrogen and oxygen—but the electrophoretic driving force inside the chambers was substantially reduced by the Pt electrode-solution interface. The resistance of the parallel-aligned nanopore was minimal in the electrical setup. Nevertheless, due to the ~ 100 nm thickness of the membrane, the electric field inside the nanopores was as high as 6 × 10^3^ V/m from a simple calculation of voltage drop/pore length. This electric field strength was higher than that in a typical gel electrophoresis setup (~ 100 V/10 cm). Therefore, despite the large parasitic resistance at the EDL of the electrode, the electrophoretic driving force inside the nanopores was effective in transporting miRNA across the membrane.

On the other hand, from their millimeter-scale length, the electric field inside the chambers was insufficient to drive the charged molecules to the high-field nanopore region. This was a limitation in the electrical setup in this work, where the chamber volume and dimensions have been designed with a view to comparison with the conventional miRNA extraction methods. Now that the electrophoretic preparation strategy was proven valid in this work, a microfluidic structure would be an efficient improvement of the fluidic chambers to exert adequate electric field throughout the whole system and increase the migration efficiency.

To confirm the feasibility of the direct electrophoretic preparation from clinical samples, miRNA was collected from human blood serum using the electrophoretic nanofilter system and the conventional columnar method as a reference. As previously mentioned, the input sample and the output buffer volumes were fixed to 150 µl and 75 µl respectively in all experiments. In the electrophoretic trials, 2-V, 30-min condition was employed and TE buffer was used as the collection buffer.

From the sequential gel electrophoresis result in Fig. 5b, the preparations of miRNA from a clinical sample were successful in both protocols. Therefore, the electrophoretic preparation system was also capable of collecting miRNA from the serum and compatible with qRT-PCR. Further, to identify the chemical components in the collection buffer after the experiment, spectrophotometry analysis was performed for both samples (Fig. 5c). The absorbance spectrum of the directly collected miRNA exhibited a peak at 280 nm wavelength, representing the presence of proteins in the solution (Fig. 5c, upper panel). A260/280 purity of the electrophoretically prepared solution was 0.594, also corresponding to the value of protein [49]. In contrast, the absorbance spectrum of the chemically extracted miRNA solution showed no sign of protein as they were excluded and washed out during the extraction process. A260/280 purity of the conventionally extracted solution was 1.72. Instead, a peak at 270 nm was observed in the spectrum (Fig. 5c, lower panel), representing phenol residue from the lysis reagent used in the extraction protocol. Phenol is a PCR inhibitor known to degrade the DNA polymerase [50]. A 260-nm peak, indicating the presence of nucleic acids, was absent in both spectra. This observation was reasonable though, from that the typical miRNA concentration in blood serum was lower than the detection limit of the spectrometer used in this study [26, 51].

Hence, protein co-migration along with miRNA apparently existed in the electrical setup, where the nanofilter membrane was incapable of completely blocking the negatively charged proteins in the blood sera moving in the same direction as miRNA. By principle, the electrophoretic transport mechanism applied to all charged particles in the chambers, including nucleic acids, albumins, and globulins, where the last two are negatively charged proteins abundantly existing in blood serum [52]. Therefore, a fundamental solution to eliminate the proteins from transporting with nucleic acids would be separation by size. However, the differences in the diameters of nucleic acid strands (single-strand: ~ 1 nm, double-strand: ~ 2 nm) and the proteins (albumins and globulins in blood serum: 4–5 nm to < 10 nm) are only a few nanometers [53], thus the window for the separation is limited. Another limitation to the separation by size is difficulties in fabrication of nanoporous structure with uniform few-nm sized nanopores. Nevertheless, despite the imperfect separation, from Fig. 5b and the results in the section below, the electrophoretically collected miRNA was still able to be analyzed by the consecutive qPCR.

In summary, the electrophoretic miRNA collection protocol showed limitations of low miRNA transport yields and imperfect separation of the nucleic acids from proteins in clinical samples. The low yield and specificity in the electrophoretic transport were native issues in the system, where the electrochemically favorable Pt electrodes acted as a large parasitic resistance and the electrophoretic migration heavily relied on the electrical nature of the biomolecules. Nevertheless, the validity of the direct electrophoretic miRNA preparation method was still proven from the gel image of the collected miRNA from clinical serum samples.

### Direct electrophoretic miRNA preparation from human blood sera

**Fig. 6:**
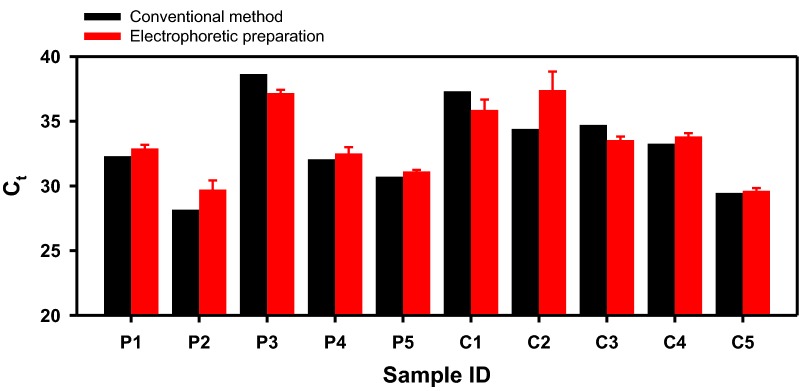
miRNA preparation from clinical human serum samples. miR93-5p was amplified from the miRNA solution collected from the sera of HCC patients (labeled as ‘P’) and the control group (labeled as ‘C’), which were prepared using the commercial Qiagen® kit (black bars) and the direct electrophoretic system (red bars). The red error bars were calculated from 3 individual electrophoretic collections

As the final demonstration of the direct miRNA preparation from clinical samples, the same procedure was conducted with the serum donated by hepatocellular carcinoma (HCC) patients and healthy individuals. In liquid biopsy studies, miR93-5p concentrations in the tissue and the blood were reported to be upregulated in diverse cancer patients including HCC compared to the control healthy group [30, 54–57]. Similar to the previous section, miRNA was prepared from each serum of 150 µl chemically (using the column-based kit) and physically (using the electrophoretic preparation system and nanofilter membrane) to 75 µl of the clean solution to proceed to qRT-PCR. The amplification results were presented in Fig. 6. As previously mentioned, the conventional method could only be performed once due to the limited amount of the sample provided, while the electrophoretic experiments were repeated 3 times. Interestingly, the C_t_ value from the electrophoretic preparation was comparable to those obtained from the conventional extractions of the same sample. The average C_t_ values from the HCC patients were 32.38 (kit extraction) and 32.69 (electrophoretic preparation) when the values from the healthy controls were 33.83 (kit) and 34.06 (electrical). Therefore, the degradation of the qPCR efficiency was small on average, or it was rather insignificant considering that the physical protocol also allowed some protein molecules to move along with miRNA. The average miR93-5p levels in the two groups were close to each other, which was an unexpected trend. One recognizable reason would be the small sample size (*n* = 5 for each group). In conclusion, the direct electrophoretic system was capable of collecting miRNA from human blood sera in both experiments, and the collected gene was identifiable and analyzable using qRT-PCR.

To summarize, the simple electrical method was successful in collecting miRNA from the clinical serum samples and was compatible with the conventional downstream application of genetic analysis, despite the impurities transported with miRNA that may have negatively affected PCR amplification.

## Conclusion

The nucleic acid preparation system based on the electrophoretic transport of the negatively charged DNA and RNA was introduced, and its performance was examined targeting the small miRNA. The new electrophoretic system was set up by a simple assembly of the components including the nanofilter membrane device with a 100-nm thickness, fabricated using top-down technique including nanoimprint. After verification of the electrophoretic transport of miR93-5p and optimization of the voltage, the system was applied in miRNA collection from human blood serum. The electrophoretic miRNA preparation from the clinical serum samples was successful in that the miRNA levels in the sera were detectable and analyzable using the new system.

Given that the operation was simple, handy, and portable, the electrophoretic strategy demonstrated its effectivity and significance as a new means of nucleic acid preparation. As discussed in the introduction, the conventional extraction method has clear limitations of complexity in operation, though the protocol is well-established in laboratories and the market. On the other hand, the electrophoretic protocol introduced in this work could be completed only in 30 min, when the columnar method required 1–1.5 h to obtain the eluted miRNA. In addition, the new method has several practical advantages over the conventional counterpart: centrifuge-free, portable using small batteries as the DC power source, and less relying on the skills of operators. The time-efficient and simple process showed comparable performances to those of the columnar kit. Therefore, the new electrophoretic protocol has potential as an easy-access on-site nucleic acid preparation tool. Even though the electrical system possessed inherent limitations such as insufficient sieving of proteins, the simple and physical protocol to collect DNA and RNA clearly worked, and even challenging tasks such as miRNA liquid biopsy from clinical blood serum could be performed using the electrophoretic isolation system.

## Data Availability

The datasets used and/or analyzed during the current study are available from the corresponding author on reasonable request.
